# Meta-Analysis and Systematic Review of Primary Renal Tubular Acidosis in Patients With Autoimmune Hepatitis and Alcoholic Hepatitis

**DOI:** 10.7759/cureus.15287

**Published:** 2021-05-28

**Authors:** Eyad Gadour, Tamer Mohamed, Zeinab Hassan, Abdalla Hassan

**Affiliations:** 1 Gastroenterology and Hepatology, University Hospitals of Morecambe Bay NHS Foundation Trust, Lancaster, GBR; 2 Acute Internal Medicine, Blackpool Teaching Hospitals NHS Foundation Trust, Blackpool, GBR; 3 Faculty of Medicine, The National Ribat University, Khartoum, SDN; 4 Medicine, Stockport Hospital NHS Foundation Trust, Manchester, GBR

**Keywords:** renal tubular acidosis, end-stage liver disease, autoimmune hepatitis, liver cirrhosis, renal failure

## Abstract

Renal and hepatic functions are often mingled through both the existence of associated primary organ diseases and hemodynamic co-relationship. The primary objective of this study was to sum up the relationship between autoimmune hepatitis (AIH) on renal tubular acidosis (RTA) and the stages of the disease. A systematic review was performed for 24 trials. A total of 3687 patients were included. The incidence of RTA occurring and short-term mortality reduction was seen in two groups; for an overall effect: Z = 2.85 (P = 0.004) a total 95% CI of 0.53 [0.34, 0.82]. Only one patient with alcoholic liver cirrhosis was found to have an incomplete type of RTA. Test for overall effect: Z = 2.28 (P = 0.02) 95% CI of 2.83 [1.16, 6.95]. A reduction in fatal infections with dual therapy of corticosteroid plus N-acetylcysteine (NAC) test for overall effect: Z = 3.07 (P = 0.002) with 95% CI of 0.45 [0.27, 0.75]. Autoimmune diseases are the most frequent underlying cause of secondary RTA in adults. The primary renal disease must be actively excluded in all patients with hepatic failure by aggressive clinical and laboratory evaluations.

## Introduction and background

Introduction

Renal dysfunction frequently accompanies advanced hepatic failure, and renal tubular acidosis (RTA) more frequently develops in case of chronic diseases of inflammatory-immunological origin [1]. One physiological consequence of deteriorating hepatic function is intrarenal vasoconstriction, which, if severe, may lead to ischemic renal injury. Ischemic renal disorders also may develop during the course of various liver diseases, such as viral hepatitis, alpha 1-antitrypsin deficiency, or autoimmune hepatitis. Furthermore, metabolic or structural diseases of the liver or kidney may cause dysfunction of the other organs (e.g., primary hyperoxaluria, methylmalonic acidemia, alpha 1-antitrypsin deficiency, tyrosinemia, and polycystic kidney disease), endotoxins, hypoxic injury, and lipid peroxidation [2], which in turn causes liver inflammation ranging from fibrosis to necrosis depending on the severity of the condition. Distal RTA or type 1 RTA is the classical form of RTA char­acterized by a failure of acid secretion by the alpha-interca­lated cells of the cortical collecting duct leading to the inability to acidify the urine that may be hereditary or may be triggered by an autoimmune disorder [3]. The body temporarily buffers hydrogen ions with plasma proteins, hemoglobin, and bicarbonate (HCO_3_), but hydrogen ions must be excreted to prevent acidosis.

The major functions of the kidneys in acid-base homeostasis are to excrete hydrogen ions and reabsorb HCO_3_. Failure to perform these functions results in HCO_3_ wasting, leading to RTA, which is categorized into three major groups: distal (type I), proximal (type II), and hyperkalemia (type IV) RTA [4]. On the other hand, chronic hepatitis, the clinical presentation, is the same as cirrhosis of the liver. The prognosis is grave if left untreated, but even in treated cases, the survival rate is not encouraging [5]. The combination of liver disease and renal dysfunction can occur as a result of systemic conditions that affect both the liver and the kidney, although primary disorders of the liver complicated by renal dysfunction are much more common. Renal failure secondary to liver dysfunction is generally prerenal and unaccompanied by alterations in renal histology, although intrinsic renal abnormalities can further complicate the acute or chronic liver disease. Postrenal acute renal failure develops rarely in chronic liver disease [6-8]. Hyponatremia or hypokalemia disorders are commonly found in patients with chronic liver disease and have been suggested as etiological agents in the sporadic cases of RTA reported in association with liver disease [9]. Autoimmune liver disease and RTA are frequently associated with hyperglobulinemia and the presence of non-organ-specific autoantibodies, and it has been suggested that auto-allergic mechanisms are involved in the pathogenesis of both disorders [8,10].

Approaching the disease, the choice between monotherapy or dual therapy and which treatment regime to choose from is a difficult one. Many published study favors corticosteroid, but others advocate the use of pentoxifylline (PTX) [11], and a few advise N-acetylcysteine (NAC) [12]. There is still disagreement on the choice of pharmacological interventions for increasing the survival rate as well as safety. This study is a review to determine the frequency with which RTA occurs in autoimmune liver disease.

Method

Systematic literature review and search were performed in all the available online medical databases such as PubMed, Embase, MEDLINE, and Cochrane Central for Controlled Trials. A protocol to include and exclude studies was already in place.

Search Methodology

This systematic review and meta-analysis were accounted for as per the PRISMA (Preferred Reporting Items of Systematic Reviews and Meta-Analysis) explanation [13]. Medical database electronic search was independently conducted by three authors; keywords such as “autoimmune hepatitis, RTA, alcoholic hepatitis,” their equivalent words, or related words were looked up in a few databases namely MEDLINE through PubMed, Scopus, Cochrane Library, and Web of Science. No filter was applied for restriction of time of publication, language, or region, but a filter for the humans-only study was applied. A manual search was done for missing data and studies that could provide references for meta-analysis.

Search Strategy

**Figure 1:**
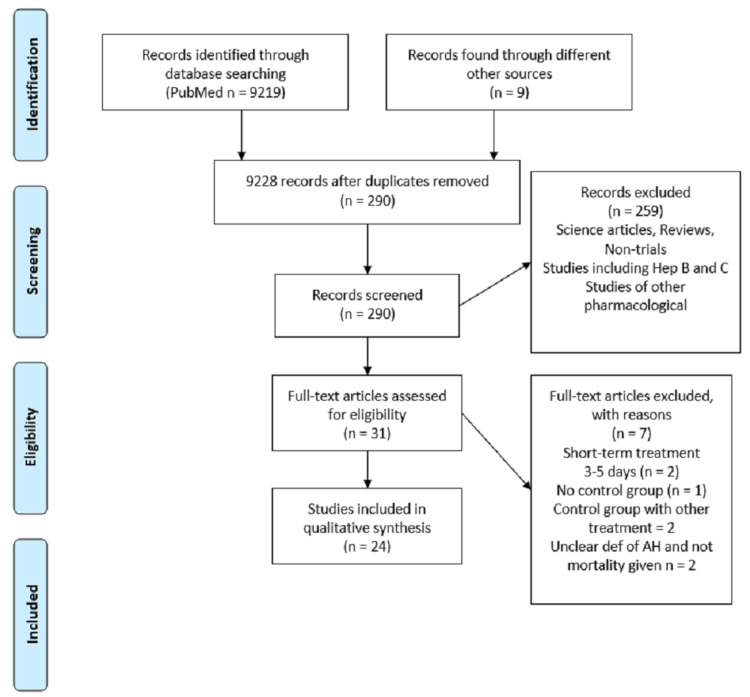
Prisma flow diagram

Electronic databases were searched for relevant terms, and all major databases such as PubMed, Cochrane, and Medline were used. A total of 9219 search results were generated with the term “alcoholic hepatitis.” Filters for human studies and clinical trials were applied, and after removing the duplicates and non-related articles, 290 studies remained. Rest were screened according to the treatment used, and all of the studies that were about autoimmune liver disease and RTA were thoroughly studied. The studies that fulfill the inclusion and exclusion criteria are shown in Figure 1.

Selection Criteria

Two authors, through combined collaboration, established inclusion and exclusion criteria, where the treatment drug, dosage, monitored outcome, and the follow-up period were taken into account. Trials were included only if they had the following:

 1. Consenting adults

 2. A clear diagnosis of autoimmune hepatitis, RTA, alcoholic hepatitis, based on biopsy, Child-Pugh score, or clinical and laboratory findings that prove autoimmune hepatitis, i.e., presence of Mallory bodies and hepatic encephalopathy [14]

 3. Therapeutic trials with follow-up regarding mortality and/or adverse effects of the target drugs

The nonhuman trials and the proven nonsignificance trials such as colchicines and ELAD were excluded, along with the trials where the treatment time period was less than a week.

Data Assessment

Studies included were independently reviewed and agreed upon by two authors, and any uncertainty was decided by a third impartial researcher. Cochrane risk-of-bias assessment tools and protocols were implied, and it was ensured that there would be no selection bias.

Data Extraction and Assessment of Quality

The selected articles were thoroughly reviewed and assessed for bias according to the Cochrane guidelines for the Cochrane hepato-biliary group module. Cochrane risk-of-bias tool for randomized trials was used, and all the studies were subjected to quality assessment for risk of bias through eight categories based on study characteristics, random sequence generation, allocation concealment, blinding of participants and personals, blinding of the outcome, incomplete outcome data, selective reporting, and other bias [15]. The quality of evidence was rated as high, moderate, low, and very low unclear.

Statistical Analysis

Direct meta-analysis was performed in the pairwise method. Four separate analyses were performed using RevMan v5.3 (Nordic Cochrane Centre, The Cochrane Collaboration, Copenhagen, 2014).

Mantel-Haenszel model was used in this statistical analysis; the studies have similar characteristics in terms of the primary and secondary outcomes of short-term mortality and occurrence of adverse events, and a 95% confidence interval was calculated among the studies and a comparison was done with other studies.

## Review

Results

A total number of 31 randomized controlled trials (RCTs) were identified by thorough online search, but seven were removed for the final analysis, the reason being the short duration of treatment for two studies (3-4 days), which were not long enough for the therapeutic effect of corticosteroids [16,17]; no comparison group in one study - both control and placebo groups received prednisolone and addition of enteral nutrition in the control group [18]; two studies had ineffective treatment for comparison arms, and dosage given was not clear and absence of placebo group [18,19]; and two studies had a vague definition of alcoholic hepatitis, and authors included mild cases of alcoholic hepatitis without histological confirmation [20].

**Figure 2:**
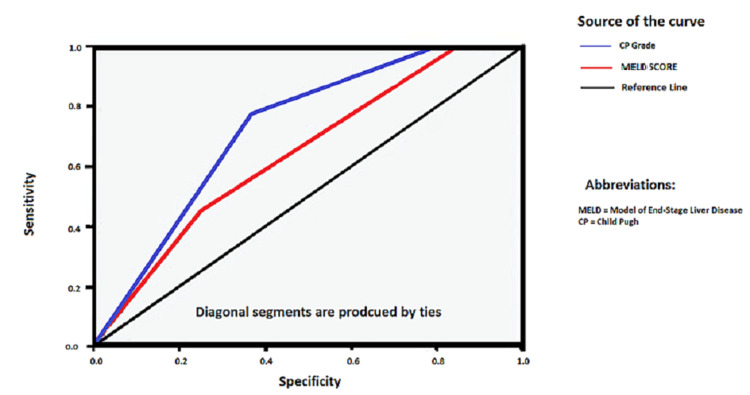
Inpatient mortality or prolonged hospitalization in correlation to Child-Pugh score

The basic information and treatment differences between incidences of RTA occurring in acute hepatitis and autoimmune hepatitis patients are shown in Table 1. The characteristics of all patients recruited in our included studies are mentioned in Table 2 in detail. Receiving-operating-characteristic (ROC) curve and the derived C-statistic provide a global and standardized appreciation of the accuracy of a marker or a composite score for predicting an event. This statistic allows a simple comparison of the accuracy of different prognostic scores within the same population. In this study, C-statistics for prediction of in-hospital mortality or prolonged hospitalization by the Child-Pugh score ranged from 0.91 to 0.63 (P = 0.021) as shown in Figure 2.

**Table 1:** Basic information and treatment differences between incidences of renal tubular acidosis occurring in autoimmune liver disease patients IQR, Interquartile range.

Variables	Decompensated Patients With Cirrhosis (n = 456)	Loss of Follow-Up for 28 Days (n = 31)	P Value	Loss of Follow-Up for 90 Days (n = 77)	P Value	Loss of Follow-Up for 6 Months (n = 158)	P Value
Sex (male), n(%)	344 (75.4%)	21 (67.7%)	0.339	54 (70.1%)	0.322	116 (73.4%)	0.614
Age, median (IQR)	53.5 (46-63.75)	56.5 (58-67.5)	0.749	75.5(55.5-68.5)	0.562	57 (56.5-68.5)	0.657
Cause of liver cirrhosis, n(%)			0.341		0.128		0.171
Viral	276 (60.5%)	21 (67.7%)		50 (71.4%)		95 (65.2%)	
Alcoholic	72 (15.8%)	7 (25.8%)		17 (22.1%)		36 (26.0%)	
Combined alcohol and viral	37 (8.1%)	2 (6.5%)		5 (3.9%)		12 (4.4%)	
Other	28 (6.1%)	1 (3.2%)		2 (2.3%)		6 (3.2%)	
Cryptogenic	43 (9.4%)	0 (0%)		2 (2.3%)		9 (1.9%)	
Cause of hospitalization, n(%)			0.897		0.394		0.094
Ascites	3 (0.7%)	0 (0%)		0 (0%)		1 (0.6%)	
Gastrointestinal bleeding	407 (89.2%)	28 (90.3%)		72 (93.5%)		151 (95.6%)	
Hepatic encephalopathy	22 (4.8%)	2 (6.5%)		3 (3.9%)		4 (2.5%)	
Infection	24 (5.3%)	1 (3.2%)		1 (3.2%)		2 (1.3%)	
Ascites degree			0.954		0.344		0.179
No ascites	195 (42.8%)	14 (45.2%)		40 (51.9%)		79 (50.0%)	
First degree	123 (27.0%)	9 (29.0%)		21 (27.3%)		48 (30.4%)	
Second degree	80 (17.5%)	5 (16.2%)		9 (11.7%)		19 (12.3%)	
Third degree	58 (12.7%)	3 (9.7%)		7 (9.1%)		12 (7.6%)	
Acute renal failure, n(%)	20 (4.4%)	1 (3.2%)	0.758	2 (2.6%)	0.466	5 (3.2%)	0.503
Hepatocellular carcinoma, n(%)	56 (12.3%)	3 (9.7%)	0.667	11 (14.2%)	0.624	26 (16.5%)	0.184
Therapy, n(%)							
Vasopressor support	144 (31.6%)	11 (35.4%)	0.239	16 (20.7%)	0.333	31 (19.6%)	0.105
Mechanical ventilation	27 (5.9%)	3 (907%)	0.400	3 (3.9%)	0.476	5 (3.2%)	0.179
Renal replacement therapy	2 (4.4%)	1 (3.2%)	0.179	1 (3.2%)	0.374	1 (0.6%)	0.763

**Table 2:** The characteristics of all patients recruited in our included studies CG, Clinical grounds; BG, biological grounds (jaundice, hepatomegaly, anorexia, transaminase level); HG, histological grounds (neutrophil, leukocyte infiltration, Mallory bodies or steatosis); MDF, Maddrey's discriminant function; Hep En, hepatic encephalopathy; GAHS, Glasgow Alcoholic Hepatitis Score; AKI, acute kidney injury; TID, three times a day

Study Characteristics	Study Design	Dx Confirmation	Intervention	Comparative Group	Follow-Up Period	Reported Results
Cabré et al., 2000 [20]	Multiple-center, open-label RCT	CG, BG, HG, MDF, Hep En	Predniosolone 40 mg/d x 28 days	Enteral nutrition	1 and 6 months	Short- and medium-term survival, incidence of fatal infections
Lebrec et al., 2010 [23]	Multiple-center, double-blind RCT	Biopsy confirmed	Prednisolone 40 mg/d for 28 days + Pentoxifylline 400 mg/d for 28 days	Prednisolone 40 mg/d and placebo for 28 days	2 and 6 months	Short- and medium-term survival rate
Ramond et al., 1992 [27]	Multiple centers, double-blind RCT		Prednisolone 40 mg/d or methylprednisolone 32 mg/d for 28 days	Placebo	1 and 3 months	Short- and medium-term survival, incidence of fatal infections
Blitzer et al., 1977 [28]	Single-center, double-blind controlled trial		Prednisolone 40 mg/d x 14 days, with taper	Placebo	28 days	Short-term survival, incidence of fatal infections
Maddrey et al., 1978 [29]	Single-center, double-blind, placebo-controlled trial	Biopsy and CG + BG	Prednisolone 40 mg/d x 28 days	Placebo	28 days	
Lesesne et al., 1978 [30]	Single-center, open label, no placebo, RCT	CG and BG, Hep En	Prednisolone 40 mg/d x 30 days	Enteral nutrition	1 month	Short-term survival, incidence of fatal infections
Depew et al., 1980 [31]	Single-center, double-blind, placebo-controlled RCT	CG + 100 Spontaneous Hep En	Prednisolone 40 mg/d x 28 days	Placebo	28 days	Short-term survival, incidence of AKI, and incidence of infections
Helman et al., 1971 [32]	Single-center, double-blind, placebo-controlled RCT	Biopsy confirmed	Prednisolone 40 mg/d x 28 days	Placebo	3 months	Short- and medium-term survival
Carithers et al., 1989 [33]	Multiple centers, double-blind, placebo-controlled RCT	CG Spon, Hep En, or MDF.32	Methylprednisolone 32 mg/d x 28 days	Placebo	28 days	Short-term survival and incidence of infections
Thursz et al., 2015 [34]	Multiple centers, double-blind, placebo-controlled RCT	CG + MDF > 32	Prednisolone 40 mg/d x 28 days	Placebo	1-3 months	Short- and medium-term survival, incidence of infections
Bories et al., 1987 [21]	Single-center, open label, no placebo, randomized trial	Biopsy and CG + BG	Prednisolone 40 mg/d x 28 days	Nutrition	1-3 months	Short-term survival
Akriviadis et al., 2000 [35]	Single-center, double-blind RCT	CG, BG, MDF, and/or Hep En	Pentoxifylline 400 mg 3x PO for 4 weeks	Placebo	1 and 6 months	Short-term survival and incidence of infections
Sidhu et al., 2012 [36]	Single-center, open-label RCT	CG, BG, and MDF > 32	Pentoxifylline 400 mg 3x PO for 4 weeks	Placebo	1 and 6 months	Short-term survival and incidence of fatal infections
Paladugu et al., 2006 [37]	Single-center, open-label RCT	CG, BG, and/or MDF > 32	Pentoxifylline for 4 weeks	Placebo	1 month	Short-term survival and incidence of infections
Moreno et al., 2010 [38]	Multiple-center, single-blind RCT	Biopsy confirmed	N-Acetylcysteine 300 mg/kg/D x 14 days	Placebo	1 and 6 months	Short- and medium-term survival, incidence of adverse effects with infections
Stewart et al., 2007 [39]	Single-center, double-blind RCT	CG, BG, and biopsy	N-Acetylcysteine 150 mg/kg/ and 100 mg/kg for 7 days	Placebo	6 months	Medium-term mortality
Garcia et al., 2012 [40]	Multiple-center, open-label RCT	CG and BG and MDF > 32	Prednisolone 40 mg/d for 28 days	PTX 400 mg TID-28 days	1 month	Short-term survival and incidence of infections
De et al., 2009 [41]	Single-center, open-label RCT	CG and BG and MDF > 32	Prednisolone 40 mg/d for 28 days	PTX 400 mg TID-56 days	1 and 6 months	Short- and medium-term survival with the incidence of infections
Park et al., 2014 [24]	Multiple-center, open-label RCT	CG and BG and MDF > 32	Prednisolone 40 mg/d for 28 days	PTX 400m g TID-28 days	1 and 6 months	Short- and medium-term survival with the incidence of infections
Thursz et al., 2015 [34]	Multiple-center, double-blind RCT	CG and BG and MDF > 32	Prednisolone 40 mg/d for 28 days with placebo	PTX 400 mg TID-28 days + Placebo, PTX 400 mg TID + Prednisolone 40 mg/d 28 days, Placebo + placebo	1 and 3 months	Short-term survival and incidence of infections
Glavind et al., 2017 [42]	Single-center, open-label, controlled Trial	CG and BG, with GASH + C43 > 9 (for severe only)	Prednisolone 40 mg/d for 14 days	PTX 400 mg TID-14 days	1 month	Short-term survival
Higuera-de et al., 2015 [7]	Single-center, open-label, randomized clinical trial	CG and BG and MDF > 32	Prednisolone 40 mg/d for 30 days	PTX 400 mg TID-30 days	1 and 3 months	Short-term survival and incidence of infections
Philips et al., 2005 [19]	Single-center, open-label RCT	CG and BG with MDF > 32	Prednisolone 30 mg/d for 28 days	N-Acetyl cysteine 150 mg/kg/d	1 month and 1 year	Short- and long-term survival with the incidence of infections
Sidhu et al., 2012 [36]	Single-center, open-label RCT	CG, BG, and MDF > 32	Prednisolone 40 mg/d for 28 days + Pentoxifylline 400 mg/d for 28 days	Prednisolone 40 mg/d and placebo for 28 days	1 and 6 months	Short-term survival and incidence of fatal infections
Mathurin et al., 2013 [43]	Multiple-center, double-blind RCT	Biopsy confirmed	Prednisolone 40 mg/d for 28 days + Pentoxifylline 400 mg/d for 28 days	Prednisolone 40 mg/d and placebo for 28 days	1 and 6 months	Short- and medium-term survival with the incidence of infections
De et al., 2014 [44]	Single-center, open-label RCT	CG, BG, and MDF > 32	Prednisolone 40 mg/d +PTX 400 mg/d for 28 days and continued	Pentoxifylline 400 mg TID for 28 days + placebo	1 month to 1 year	Short- and long-term mortality with the incidence of infections
Nguyen-Khac et al., 2011 [45]	Multiple-center, open-label RCT	CG, BG, and MDF > 32	Prednisolone 40 mg/d for 28 days + N-acetylcysteine x 5 days	Prednisolone 40 mg/d for 28 days + placebo for 5 days	1 month and 6 months	Short- and medium-term mortality with the incidence of infections

Characteristics of included studies

The studies were categorized in a pair-way scheme for a direct statistical analysis; the main objective was to analyze each study discussing autoimmune hepatitis: RTA and different stages of the disease and acidosis. Following this pattern, we had autoimmune hepatitis (Group 1); RTA (Group 2); and steroids, N-acetylcysteine, and pentoxifylline versus placebo (Group 3). Group 2 comprised of a comparison of drugs with each other, i.e., corticosteroids versus PTX and corticosteroids versus N-acetylcysteine; Group 3 constitutes the combination therapy versus placebo or a single drug. A total of 3687 patients were included in total subgroups, although few fell into different categories of the same treatment group due to similarities in comparative groups.

As there was no limitation applied in inclusion criteria for the date of publication, the patients included in this study were diagnosed with autoimmune hepatitis mainly on clinical grounds [21] and prolongation of prothrombin time for five to nine seconds than control. Maddrey’s D45F ≥ 32 was used as the definition of severe alcoholic hepatitis. The duration of follow-up was ranging from one month to one year, but multiple studies were terminated after two to three months. The common exclusion was made if patients had an episode of gastrointestinal bleed in a week prior to the start of therapy or before randomization also if hepatitis was concomitant with any of the viral etiologies such as hepatitis B or C26. The included studies were mostly single-center trials, but few were multiple-center simultaneous trials, especially the studies [22-26]. All studies show heterogeneity in the participants, although it was graded very low in some older studies and in Garcia where only four female participants were included. The characteristics of included studies are given in Table 2.

Mortality

Short-term mortality was analyzed according to the treatment regimes, and all the studies that had a follow-up of three to six months were analyzed for medium-term mortality with a period ranging from two months to one year.

**Figure 3:**
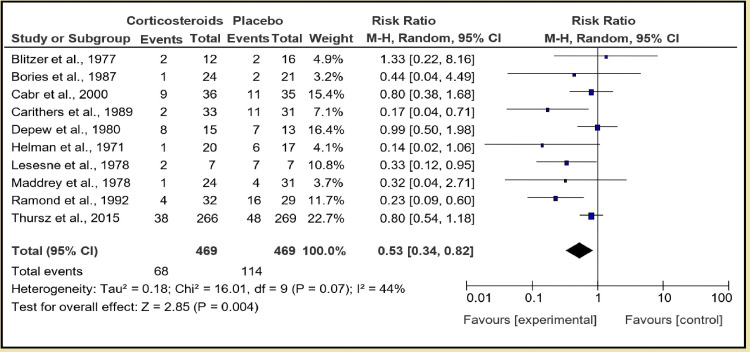
Group 1, Section 1 (short-term mortality)

A total number of 11 studies were categorized in the first section of this group. Corticosteroid in the form of prednisolone or methylprednisolone given at 40 mg or 32 mg, respectively, versus placebo, pooled analysis of 940 patients with 471 in corticosteroid group and 469 in placebo group shows that corticosteroids are more effective than placebo in decreasing short-term mortality with an overall effect of 0.53, with a positive Z score [95% CI of 0.34, 0.82]. Test for overall effect: Z = 2.85 (P = 0.004), and heterogeneity: Tau² = 0.18; Chi² = 16.01, df = 9 (P = 0.07); I² = 44% (Figure 3).

**Figure 4:**
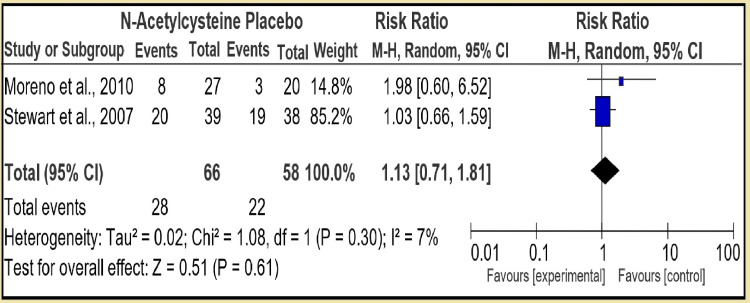
Group 1, Section 2. N-Acetylcysteine vs Placebo

**Figure 5:**
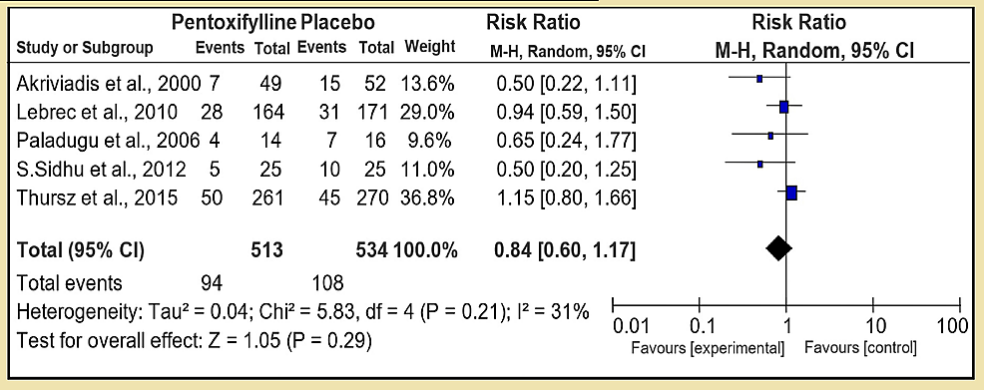
Group 1, Section 3. Pentoxifylline vs Placebo

In the second section, we compared N-acetylcysteine to placebo with two RCTs and pooled number of 66 controls to 58 placebo patients. The therapy had test for overall effect: Z = 0.51 (P = 0.61) with risk ratio (RR) 1.13 [95% CI, 0.71, 1.81], which was not statistically significant with low heterogeneity: Tau² = 0.02; Chi² = 1.08, df = 1 (P = 0.30); I² = 7% (Figure 4). However, in the third section of first group, pentoxifylline was compared to placebo (5 RCT, RR 0.84 [95% CI, 0.60, 1.17]) and test for overall effect: Z = 1.05 (P = 0.29) showing pentoxifylline is statistically not superior to placebo: Tau² = 0.04; Chi² = 5.83, df = 4 (P = 0.21); I² = 31%, and the heterogeneity was low (Figure 5).

**Figure 6:**
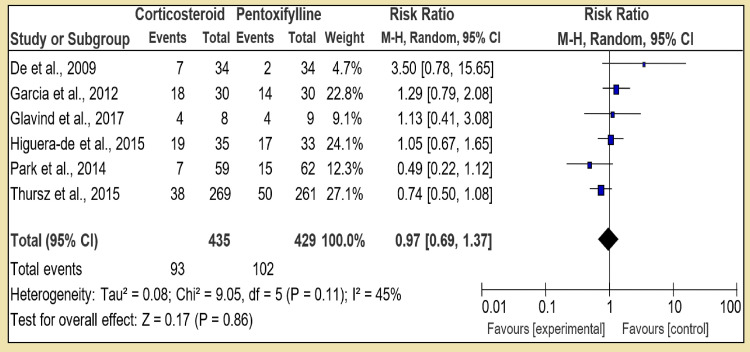
Group 2, Section 1. Corticosteroid vs Pentoxifylline

**Figure 7:**
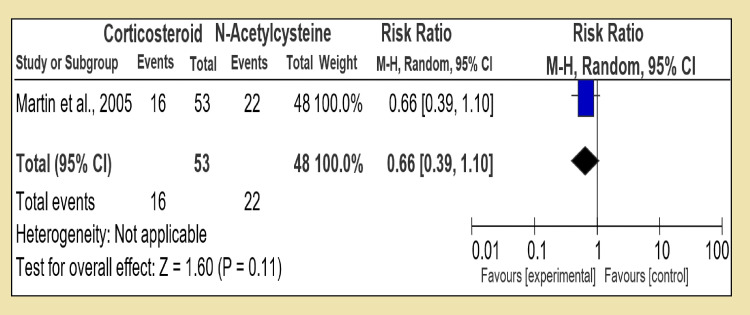
Group 2, Section 2. Corticosteroid vs N-Acetylcysteine

However, in the first section of Group 2, corticosteroid was compared to pentoxifylline alone with six RCTs, and the pooled patients in the corticosteroid group were 435 against pentoxifylline which was 429; the direct comparison showed that test for overall effect: Z = 0.17 (P = 0.86). The low Z value signifies that corticosteroids are not superior to pentoxifylline when given in monotherapy; heterogeneity: Tau² = 0.08; Chi² = 9.05, df = 5 (P = 0.11); I² = 45%, while heterogeneity among these trials was statistically significant (Figure 6). In the second section of Group 2, corticosteroid was compared with N-acetylcysteine; both were given monotherapy. There was only one RCT found for it conducted by Martin in 2005. With 16 patients in the corticosteroid group and 22 in the N-acetylcysteine group, the study favors that corticosteroid was statistically insignificant. With test for overall effect: Z = 1.60 (P = 0.11) and RR 0.66 [95% CI, 0.39, 1.10]; being single study, heterogeneity was not applicable (Figure 7).

However, in the third group (Group 3), a combination of dual therapies was compared with a single therapy. In the first section, we compared a combination of corticosteroid and pentoxifylline with corticosteroid alone; in four RCTs, we had statistically insignificant data of test for overall effect: Z = 0.39 (P = 0.70) showing that in terms of increasing short-term survival, both therapies have the same effect RR 0.94 [95% CI, 0.68, 1.29]. However, in the second section of Group 3, corticosteroid in combination with pentoxifylline versus pentoxifylline alone was compared, and two RCTs were analyzed with the test for overall effect: Z = 1.96 (P = 0.05) with RR 0.68 [95% CI, 0.46, 1.00]. The third section compared corticosteroid with N-acetylcysteine versus corticosteroid alone; this section included only one RCT with the test for overall effect: Z = 2.57 (P = 0.01) showing corticosteroid therapy alone is superior in decreasing short-term mortality of alcoholic hepatitis with RR 0.35 [95% CI, 0.16, 0.78].

In the first section of Group 1, a comparison was made between corticosteroid and placebo monotherapy; six RCTs were included as others did not have a follow-up. The test for overall effect: Z = 1.31 (P = 0.19) with RR 0.57 [95% CI, 0.25, 1.32]. In the second section, pentoxifylline was compared to placebo, and the test for overall effect was: Z = 0.76 (P = 0.45) with RR 0.86 [95% CI, 0.59, 1.27], whereas in the third section, N-acetylcysteine versus placebo, only one RCT had follow-up data available and test for overall effect: Z = 0.14 (P = 0.89) with RR 1.06 [0.49, 2.29] was statistically insignificant. Overall, all three sections represented that only corticosteroid monotherapy was favorable with a slight increase in the medium-term mortality; other sections provided insignificant results.

We have corticosteroid versus pentoxifylline for the direct comparison of two therapies in the first section of Group 2. A total of four RCTs were available with sufficient data. Test for overall effect: Z = 1.58 (P = 0.11) with RR of 1.40 [0.92, 2.12] suggesting a slight but statistically insignificant tilt in favor of pentoxifylline. In the second section, corticosteroid vs N-acetylcysteine was compared, but only one RCT was available. Test for overall effect: Z = 1.28 (P = 0.20) with RR 0.81 [95% CI, 0.59, 1.12].

**Figure 8:**
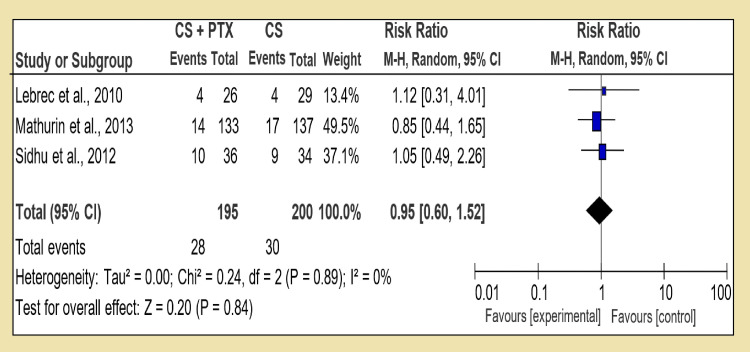
Group 3, Section 1. Corticosteroid + Pentoxifylline vs Corticosteroid alone

**Figure 9:**
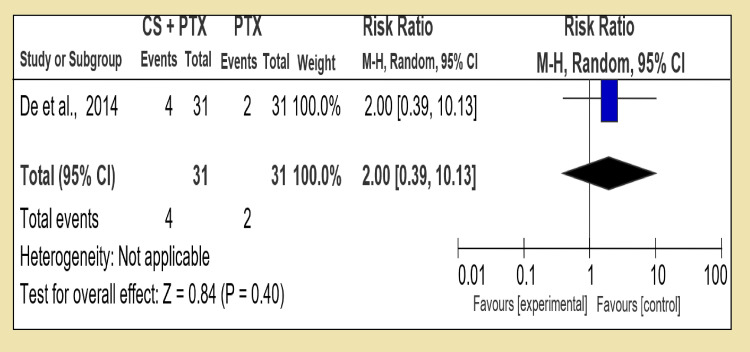
Group 3, Section 2. Corticosteroid + Pentoxifylline vs Pentoxifylline

**Figure 10:**
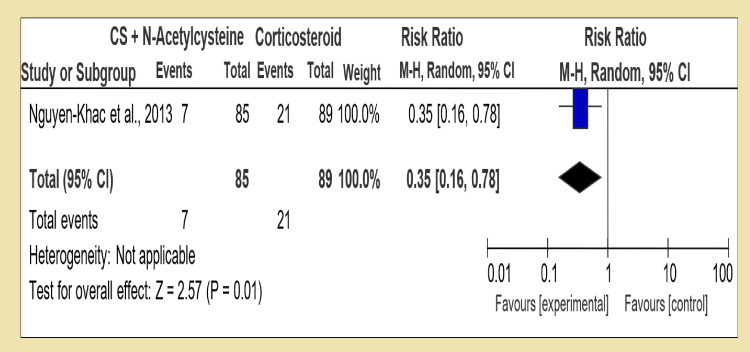
Group 3, Section 3. Corticosteroid + N-Acetylcysteine vs Corticosteroid alone

By comparing combination treatment with the single one (Group 3), we analyzed corticosteroid and pentoxifylline with corticosteroid alone and RR 1.01 [95% CI, 0.60, 1.52], and test for overall effect: Z = 0.20 (P = 0.84) was available, which was inconclusive (Figure 8). Only one RCT was available to compare corticosteroid and pentoxifylline versus PTX, and it shows test for overall effect: Z = 0.84 (P = 0.40) and RR 1.67 [95% CI, 0.39, 10.13] (Figure 9). Interestingly in last section of Group 3, corticosteroid and N-acetylcysteine against corticosteroid alone, the test for overall effect: Z = 2.57 (P = 0.01) and RR 0.88 [95% CI, 0.16, 0.78] (Figure 10). The difficulty in comparing two regimes for medium-term mortality was mainly due to loss of subjects or high occurrence of fatal infections, which meant a loss of compliance.

Secondary objective

Risk of Fatal Hemorrhage

**Figure 11:**
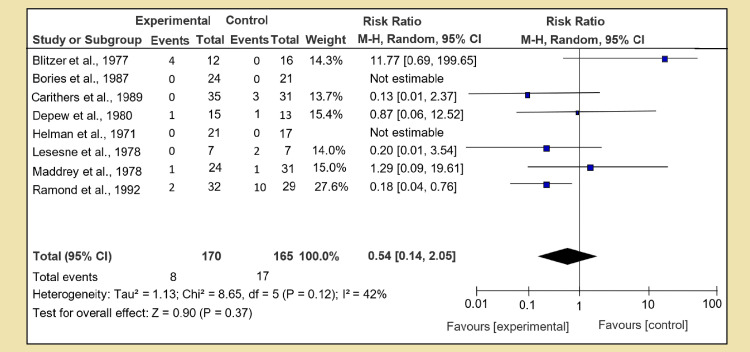
Secondary outcome A: risk of fatal hemorrhage

The risk of fatal hemorrhage was calculated and analyzed in the same fashion of pairwise direct analysis, and for Section 1 of Group 1, we compared the corticosteroid versus placebo. Data from a couple of studies were missing; eight RCTs were analyzed, and test for overall effect: Z = 0.50 (P = 0.37) was calculated with RR of 0.54 [95% CI, 0.24, 1.21]. No strong link was found among the corticosteroid and placebo group, though heterogeneity was very low as shown in Figure 11. Heterogeneity: Tau² = 0.00; Chi² = 4.43, df = 6 (P = 0.62); I² = 0%. In the same group, Section 2, we calculated the risk with pentoxifylline when compared against placebo. Only one RCT was available with test for overall effect: Z = 0.14 (P = 0.89) and RR 1.06 [95% CI, 0.49, 2.29]; no data was available for N-acetylcysteine in Section 3.

In Group 2 (corticosteroid versus pentoxifylline), two RCTs were available; test for overall effect: Z = 0.68 (P = 0.50) with RR 0.73 [95% CI, 0.29, 1.83]; for chitosan (CS) against N-acetylcysteine, the P value was 0.81. Test for overall effect: Z = 0.26 [95% CI, 0.17, 3.82]. In Group 3 (corticosteroid with pentoxifylline versus PTX and CS), test for overall effect: Z = 2.28 (P = 0.02) with RR 2.83 [95% CI, 1.16, 6.95] and test for overall effect: Z = 0.40 (P = 0.69) with RR 1.33 [95% CI, 0.32, 5.47] were obtained, respectively. For Section 3 (NAC against CS), test for overall effect: Z = 1.90 (P = 0.06).

Risk of Fatal Infections

**Figure 12:**
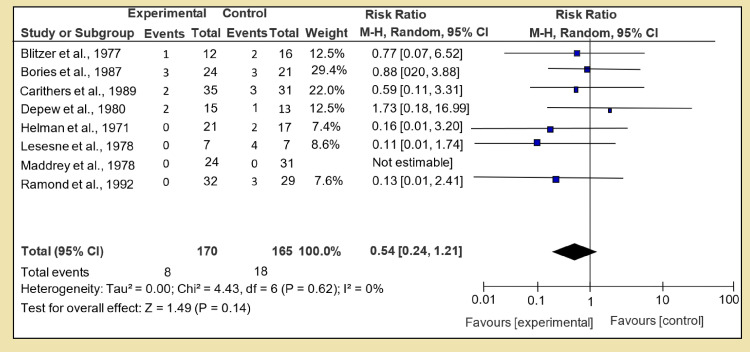
Secondary outcome B: risk of fatal infections and sepsis

The dates of all the available trials were analyzed for the risk of fatal infections, and infections occurring during the short term after or during the treatment were calculated; the data were in terms of infections and sepsis. The follow-up of older studies and a few newer ones were less than desirable. All the available calculations are shown in Figure 12.

Incidence of infections in steroid group was higher, but the combined data suggest it is insignificant. Test for pentoxifylline versus placebo gave overall effect: Z = 0.04 (P = 0.97) and 95% CI ranging [0.07, 16.50] with RR 1.06; for Section 3, no RCT was available. In Group 2 of CS against PTX, RR of fatal infections was 0.84 [95% CI, 0.23, 3.07] and test for overall effect: Z = 0.27 (P = 0.79), whereas for CS compared to N-acetylcysteine, test for overall effect: Z = 1.51 (P = 0.13) with RR 0.52 [0.22, 1.22] in Group 3. The CS + PTXVs CS for infections gave test for overall effect: Z = 0.11 (P = 0.91), a statistically insignificant finding. CS + PTXVS PTX test for overall effect: Z = 0.98 (P = 0.33) RR 0.33 [95% CI, 0.04, 3.03] and steroid plus N-acetylcysteine versus steroids alone gave a RR of 0.45 with 95% CI [0.27, 0.75] and test for overall effect: Z = 3.07 (P = 0.002), which not only is statistically significant but also shows a strong relation between occurrence of infections when corticosteroid and antioxidants (N-acetylcysteine) are used concomitantly. Further research in this claim is needed as it is only derived from a single RCT.

Quality of evidence

GRADE approach for network meta-analysis

The assessment of risk of bias is shown in Table 3.

**Table 3:** Assessment of risk of bias

Study Characteristics	Random Sequence Generator	Allocation Concealment	Blinding of Participants and Personnel	Blinding of Outcome	Incomplete Outcome Data	Selective Reporting	Other Bias
Cabré et al., 2000 [20]	Low	Low	Low	Low	Low	Low	Low
Ramond et al., 1992 [27]	Low	Low	Low	Low	Low	Low	Low
Blitzer et al., 1977 [28]	Low	Low	Low	Unclear	Low	Low	Low
Maddrey et al., 1978 [29]	Low	Unclear	Low	Low	Low	Low	Low
Lesesne et al., 1978 [30]	Unclear	Unclear	High	Low	Low	Low	Low
Depew et al., 1980 [31]	Unclear	Unclear	Low	Low	Low	Low	Low
Helman et al., 1971 [32]	Unclear	Low	Low	Low	Low	Low	Low
Carithers et al., 1989 [33]	Low	Low	Low	Low	Low	Low	Low
Thursz et al., 2015 [34]	Low	Low	Low	Low	Low	Low	Low
Bories et al., 1987 [21]	Low	Unclear	High	Unclear	Low	Low	Low
Pentoxifylline vs Placebo							
Akriviadis et al., 2000 [35]	Low	Low	Low	Low	Low	Low	Low
Sidhu et al., 2012 [36]	Low	Low	Unclear	Unclear	Low	Low	Low
Paladugu et al., 2006 [37]	Unclear	Unclear	Unclear	Unclear	Unclear	Unclear	High
Lebrec et al., 2010 [23]	Low	Low	Low	Low	Low	Low	Low
N-Acetylcysteine vs Placebo							
Moreno et al., 2010 [38]	Low	Unclear	Low	Low	Low	Low	Low
Stewart et al., 2007 [39]	Low	Low	Low	Low	Unclear	Low	High
Garcia et al., 2012 [40]	Unclear	Unclear	Low	Low	Low	Low	Low
De et al., 2009 [41]	Low	Unclear	Low	Low	Low	Low	Low
Park et al., 2014 [24]	Low	Low	Low	Low	Low	Low	Low
Thursz et al., 2015 [34]	Low	Low	Low	Low	Low	Low	Low
Glavind et al., 2017 [42]	High	High	High	Low	Low	Low	Low
Martin et al., 2005 [19]	Low	Unclear	Low	Low	Low	Low	Low
Sidhu et al., 2012 [36]	Low	Low	Low	Low	Low	Low	Low
Mathurin et al., 2013 [43]	Low	Low	Low	Low	Low	Low	Low
De et al., 2014 [44]	Unclear	Unclear	Low	Low	Low	Low	Low
Corticosteroids + N-Acetylcysteine vs CS							
Nguyen-Khac et al., 2013 [45]	Low	Low	Low	Low	Low	Low	Low

Discussion

Autoimmune liver disease and RTA are frequently associated with hyperglobulinemia and the presence of non-organ-specific autoantibodies, and it has been suggested that auto-allergic mechanisms are involved in the pathogenesis of both disorders [2]. Golding et al. reported that abnormalities of the serum immunoglobulin were detected in all patients with both RTA and autoimmune liver disease, but the presence of the acidification defect was not related either to the level or class of immunoglobulin elevated [46]. Doniach et al. in the 1960s concluded that with the exception of mitochondrial antibody, autoantibodies were found with equal frequency in patients with and without RTA. The increased frequency of mitochondrial antibody (72%) in the group with RTA is probably explained by the fact that this group contained a large number of cases of primary biliary cirrhosis (56%), a condition known to be associated with a high incidence of mitochondrial antibody. The pentoxifylline is also a TNF inhibitor, and its main role is hypothesized to be decreasing mortality by preventing HRS, though it is successfully used in treating vascular diseases for its property of improving blood flow and endow RBCS with increased elasticity [37,47]. The previous studies and our study showed that N-acetylcysteine is an antioxidant, and it also replenishes the glutathione (major antioxidant) in your cells; so it is assumed that its role is protective in nature [48].

From our meta-analysis, we have also concluded that corticosteroids are effective in decreasing short-term mortality, and corticosteroid monotherapy is more effective than monotherapy of pentoxifylline or N-acetylcysteine alone. But we must also consider the limitations of this therapy. Most of the trials of corticosteroids had an exclusion criterion for patients who were suffering from an infection or had an active gastrointestinal bleed. Both conditions are common in alcoholics. This, in the author’s opinion, is a point to ponder. On the other hand, pentoxifylline or N-acetylcysteine monotherapy could not provide any statistically significant proof of their effectiveness, even after the inclusion of Steroids or Pentoxyfilline in Alcoholic Hepatitis (STOPAH) trials.

In the second group of our short-term mortality comparison where monotherapies were compared by direct analysis, corticosteroid versus pentoxifylline monotherapy, a slight but insignificant tilt in corticosteroid direction can be observed. But in the author’s opinion as all these studies were performed between 2009 and 2015 and as they were comparatively of high quality, there is no doubt in their accuracy, though bias is always an issue. In the second analysis, with a P value of 0.11 and Z value of 1.60, this single RCT trial favors corticosteroid rather than N-acetylcysteine. In the third group with combination treatment of two regimes versus a single drug, no significant findings were available, except that corticosteroid in concomitant administration with N-acetylcysteine is showed to be more effective than corticosteroid alone with a Z value of 2.57, but this comparison cannot be given much weight as it originated from a single study again. The author believes if this treatment is promising, further research is warranted.

In terms of medium-term mortality that can be defined as anywhere from two months to one year, the important time period according to the author is from the second month to the sixth month as this is the compulsory waiting period for a patient to remain alcohol-abstinent to become qualified to be included in National Organ Transplant Committee for a liver transplant. The data pertaining to medium-term mortality in the first comparative group was insignificant; a decrease in the medium-term mortality was expected from the corticosteroid group, but that was only limited to short-term mortality, and in medium-term mortality, the survival corticosteroid plays a statistically insignificant role. Test for overall effect: Z = 1.31 (P = 0.19), risk ratio of 0.57 with 95% CI ranging from 0.25 to 1.32. A notable finding was the data on pentoxifylline alone which through confidence interval approached OR: Z = 0.76 (P = 0.45), which makes the author believe that with further studies and an increased pool with strict mid-term follow-up measures, we can gather more accurate data on the survival of alcoholic hepatitis patients if only pentoxifylline is given. In this section, pentoxifylline is reported to be more effective than corticosteroids in reducing short-term mortality when compared with each other. Z = 1.58 (P = 0.11); no other finding of analysis was statistically important to be discussed.

As we have mentioned, the secondary objective was to calculate the risk of fatal hemorrhage and infections leading to sepsis; the main reason to include these two adverse effects and not HRS was there is already enough data on the occurrence of hepato-renal syndrome. The alcoholic patients are prone to infections especially after the administration of treatment as their main role is suppression of immune cells to halt the inflammatory process and gastrointestinal bleeding as the liver is fibrotic or cirrhotic causes portal hypertension and varices, which are prone to rupture. Hence authors decided to choose this as a secondary objective to evaluate the incidence rate of these events after the administration of therapy and if the occurrence is lessened.

On alcoholic hepatitis, the risk of fatal hemorrhage increased when prednisone or methyl-prednisone was given if compared to placebo, but the value did not reach statistical importance. The overall effect Z = 1.49 and P = 0.14. All other analyses did not provide significant proof, but in a combination of steroid and pentoxifylline against steroids alone, the Z effect was 2. 41 and P value was 0.02, indicating that dual therapy is safer than single-drug treatment. Another worth mentioning finding was increased risk of fatal hemorrhage, which was seen in combination therapy of N-acetylcysteine with corticosteroids. The events of fatal hemorrhage were higher than corticosteroid alone. The reason why this dual therapy is more prone to fatal hemorrhage needs further investigation and trials.

The risk of Infections was also measured with each therapy. The hypothesis that corticosteroid slows down the inflammatory process also would affect the immunity of the host. But in the first direct analysis against placebo, the incidence of fatal infections or sepsis was insignificant, with a Z value of 0.09 and a P value of 0.37. Though there was moderate heterogeneity among studies 42%/, no conclusion could be drawn as confidence interval surpassed OR. Again all the trials gave insufficient proof to support this claim, but N-acetylcysteine + CS versus N-acetylcysteine alone gave a test of overall effect: Z = 3.07 and P = 0.002, but as it was based on a single study, more investigations are required to settle the debate.

## Conclusions

Autoimmune diseases are the most frequent underlying cause of secondary RTA in adults, which is similar to other studies in the literature. The primary renal disease must be actively excluded in all patients with hepatic failure by aggressive clinical and laboratory evaluations. Corticosteroid monotherapy and dual therapy of corticosteroid with N-acetylcysteine improve the short-term survival rate in autoimmune hepatitis and alcohol liver disease. Although defects of urinary acidification may arise from several different mechanisms, it is possible that renal tubular acidosis and autoimmune liver disease may form part of a systemic disorder capable of affecting many organs.
